# Severe dermatitis, multiple allergies, and metabolic wasting syndrome caused by a novel mutation in the N-terminal plakin domain of desmoplakin

**DOI:** 10.1016/j.jaci.2015.05.002

**Published:** 2015-11

**Authors:** Maeve A. McAleer, Elizabeth Pohler, Frances J.D. Smith, Neil J. Wilson, Christian Cole, Stuart MacGowan, Jennifer L. Koetsier, Lisa M. Godsel, Robert M. Harmon, Robert Gruber, Debra Crumrine, Peter M. Elias, Michael McDermott, Karina Butler, Annemarie Broderick, Ofer Sarig, Eli Sprecher, Kathleen J. Green, W.H. Irwin McLean, Alan D. Irvine

**Affiliations:** a Clinical Medicine, Trinity College Dublin, Dublin, Ireland; b Pediatric Dermatology, Our Lady's Children's Hospital Crumlin, Dublin, Ireland; c National Children's Research Centre, Our Lady's Children's Hospital Crumlin, Dublin, Ireland; j Infectious Disease Department, Our Lady's Children's Hospital Crumlin, Dublin, Ireland; d Dermatology and Genetic Medicine, University of Dundee, Dundee, United Kingdom; e Division of Computational Biology, College of Life Sciences, University of Dundee, Dundee, United Kingdom; f Department of Pathology, Northwestern University Feinberg School of Medicine, Chicago, Ill; g Department of Dermatology, Northwestern University Feinberg School of Medicine, Chicago, Ill; h Department of Dermatology and Venereology, Innsbruck Medical University, Innsbruck, Austria; i Dermatology Service, Veterans Affairs Medical Center, San Francisco, and the Department of Dermatology, University of California, San Francisco, Calif; k Department of Gastroenterology, Our Lady's Children's Hospital Crumlin and School of Medicine and Medical Science, University College Dublin, Dublin, Ireland; l Department of Dermatology, Tel Aviv Sourasky Medical Center, Tel Aviv, Israel; m Department of Human Molecular Genetics & Biochemistry, Sackler Faculty of Medicine, Tel Aviv University, Tel Aviv, Israel

**Keywords:** Atopy, skin barrier, atopic dermatitis, desmosome, desmoplakin, atopic sensitization, eosinophilic esophagitis, aCGH, Array comparative genome hybridization, AD, Atopic dermatitis, DSG1, Desmoglein 1 gene, DSP, Desmoplakin gene, PPK, Palmoplantar keratoderma, SAM, Severe dermatitis, multiple allergies, and metabolic wasting, SNP, Single nucleotide polymorphism, SPINK5, Serine protease inhibitor Kazal-type 5 gene, WES, Whole-exome sequencing

## Abstract

**Background:**

Severe dermatitis, multiple allergies, and metabolic wasting (SAM) syndrome is a recently recognized syndrome caused by mutations in the desmoglein 1 gene *(DSG1)*. To date, only 3 families have been reported.

**Objective:**

We studied a new case of SAM syndrome known to have no mutations in *DSG1* to detail the clinical, histopathologic, immunofluorescent, and ultrastructural phenotype and to identify the underlying molecular mechanisms in this rare genodermatosis.

**Methods:**

Histopathologic, electron microscopy, and immunofluorescent studies were performed. Whole-exome sequencing data were interrogated for mutations in desmosomal and other skin structural genes, followed by Sanger sequencing of candidate genes in the patient and his parents.

**Results:**

No mutations were identified in *DSG1*; however, a novel *de novo* heterozygous missense c.1757A>C mutation in the desmoplakin gene *(DSP)* was identified in the patient, predicting the amino acid substitution p.His586Pro in the desmoplakin polypeptide.

**Conclusions:**

SAM syndrome can be caused by mutations in both *DSG1* and *DSP*. Knowledge of this genetic heterogeneity is important for both analysis of patients and genetic counseling of families. This condition and these observations reinforce the importance of heritable skin barrier defects, in this case desmosomal proteins, in the pathogenesis of atopic disease.

Mendelian skin diseases that include atopic dermatitis (AD) or AD-like dermatitis, food allergies, or both as part of their phenotype potentially offer significant insights into disease pathogenesis. The best examples of insights into AD pathogenesis from Mendelian disease are Netherton syndrome (Mendelian Inheritance in Man [MIM] #256500) and ichthyosis vulgaris (MIM #146700). The discovery of mutations in the serine protease inhibitor Kazal-type 5 gene *(SPINK5)* as causative for Netherton syndrome^1^ highlighted the role of excessive stratum corneum protease activity in patients with AD, and identification of mutations in the filaggrin gene *(FLG)* in patients with ichthyosis vulgaris^2^ has been transformative in our understanding of the importance of the skin barrier in patients with AD and those with allergic sensitization.^3^, ^4^ Other rare genodermatoses, such as “type B” peeling skin syndrome, which is attributed to mutations in the corneodesmosin gene *(CSDN)*,^5^ reinforce the importance of stratum corneum cohesion in the pathogenesis of AD.^6^ In 2013, a new syndrome designated severe dermatitis, multiple allergies, and metabolic wasting (SAM) syndrome was attributed to loss-of-function mutations in the desmosomal plaque protein desmoglein 1 (desmoglein 1 gene *[DSG1])*.^7^ Four cases of SAM syndrome have now been reported, all caused by *DSG1* mutations.^7^, ^8^ Here we report the first case of SAM syndrome (MIM #615508) attributed to a missense mutation affecting the plakin domain of the desmoplakin gene *(DSP)*.

## Methods

### Clinical history

**Fig 1:**
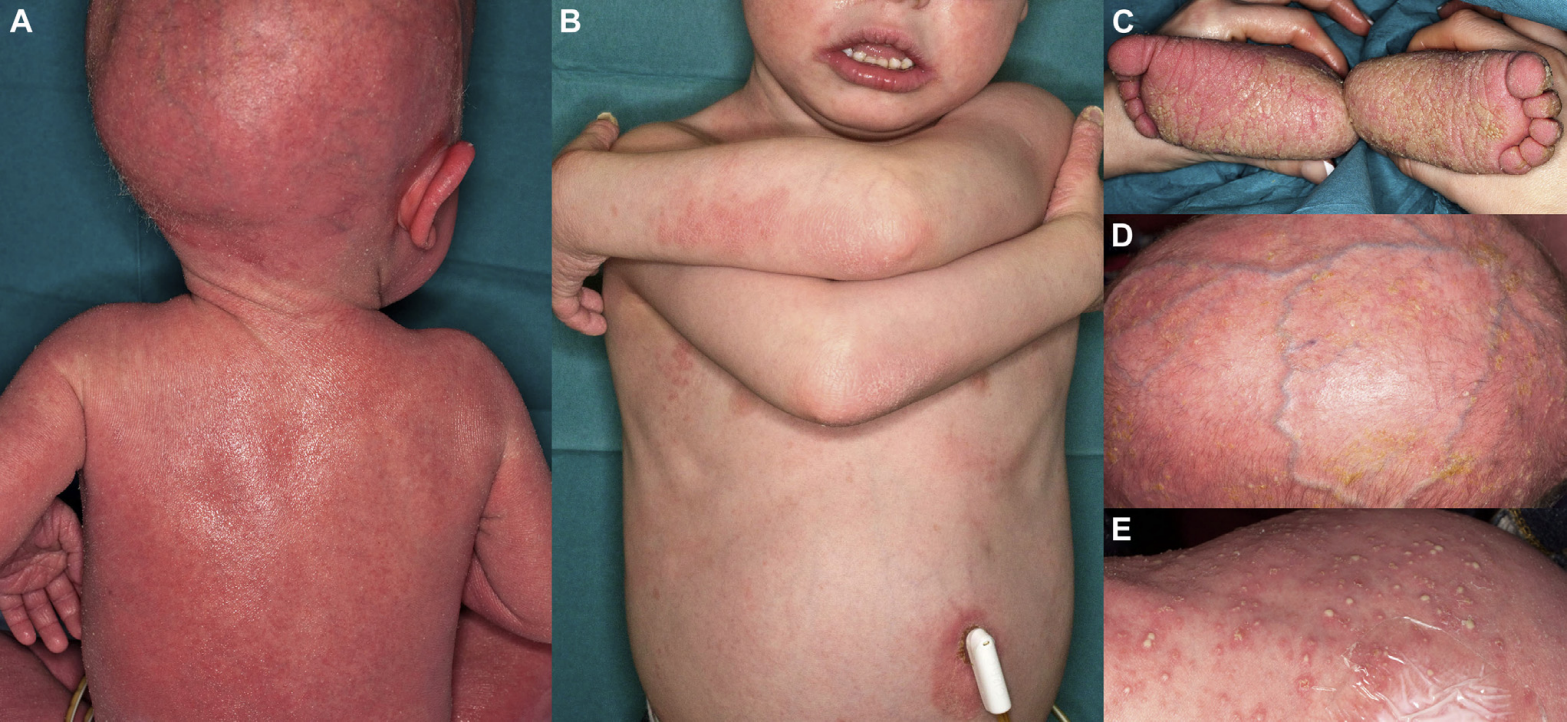
Clinical phenotype. **A,** Generalized erythroderma and ichthyosis when the patient was 6 months of age. Hypotrichosis and macrocephaly are also evident. **B,** Improvement in erythrodermic ichthyosis after treatment with acitretin. **C,** Plantar keratoderma. **D,** Erythema, scaling, and diffuse hypotrichosis of the scalp. **E,** Pustulosis on the abdomen during an episode of systemic sepsis.

We report the case of a white boy, now aged 6 years, with SAM syndrome. The patient is the first child of Irish, nonconsanguineous healthy parents. He was born at 39 weeks and 5 days' gestation by means of an emergency cesarean section because of fetal bradycardia after rupture of the membranes. He weighed 3.18 kg at birth (25th percentile). The perinatal history was suggestive of a collodion membrane. Erythroderma with “skin scaling and peeling” was reported to be present from the first weeks of life. He was referred to our department at 6 months of age. He had marked erythroderma, ichthyosis, palmoplantar keratoderma (PPK) with nail dystrophy, and diffuse hypotrichosis. (Fig 1, *A*, *C*, and *D*). Intermittently, especially with erythrodermic flares, he had a widespread superficial pustulosis (Fig 1, *E*). He also had severe and intractable itch. He was on the second percentile for weight and the fourth percentile for height. He also had macrocephaly, with his occipitofrontal circumference being greater than the 99th percentile; global developmental delay; and nystagmus.

In the first 3 years of life, the patient had repeated episodes of systemic sepsis necessitating hospital admission for intravenous antibiotics and supportive management. These septic episodes were accompanied by photophobia, with a flare in ichthyosiform erythroderma and pustulosis and an exacerbation in itch. The most frequently isolated pathogens causing sepsis were both the methicillin-sensitive and methicillin-resistant strains of *Staphylococcus aureus*. An extensive metabolic, immunologic, and infectious disease workup did not yield a specific diagnosis. The consensus opinion was that the source of infection was the patient's skin. He failed to thrive and had frequent vomiting, abdominal pain, diarrhea, and food aversion. Several therapeutic approaches to improve his nutrition failed. A percutaneous endoscopic gastrostomy tube was sited when he was 10 months of age to provide essential supplemental feeding.

Neurologic assessment confirmed mild global developmental delay. Computed tomographic brain scanning demonstrated mild dilatation of the lateral and third ventricles, with diffuse prominence of sulci over both cerebral hemispheres. The brain appeared otherwise normal. Ophthalmology assessment demonstrated keratitis. Binocular visual evoked responses and electroretinographic results were within normal limits. Despite extensive assessment, no cause of nystagmus was established. The results of cardiology workup, including echocardiography, were unremarkable.

Several therapeutic approaches to treat the patient's skin and systemic infection were tried with variable success. Potent topical corticosteroids and corticosteroid and antimicrobial combination therapy yielded minimal improvement. Topical tacrolimus ointment, with careful monitoring of tacrolimus blood levels, resulted in moderate improvement. The patient's skin was observed to improve when he received antibiotics for systemic sepsis. Prophylactic anti-staphylococcal antimicrobials had no effect. A trial of anakinra proved ineffective. Systemic acitretin (0.5 mg/kg/d) had good effect, with a significant improvement in PPK and ichthyosis. Intravenous immunoglobulin infusions were also effective in reducing the frequency of septic episodes. The combined beneficial effects of acitretin, intravenous immunoglobulin, and percutaneous endoscopic gastrostomy feeding have allowed improvement in the patient's dermatitis, growth and weight gain, and development (Fig 1, *B*). His hair started to grow when he was 6 years old and is a wooly hair phenotype. Also, he has had ongoing poor periodontal health, with marked hypodontia.

The patient was atopic, with multiple food allergies. Blood investigations were consistent with atopy, with persistent eosinophilia (0.4-0.5 × 10^9^/L) and increased IgE levels (611 kU/L). Analysis of specific IgEs confirmed sensitization to egg, peanut, and wheat, despite having no oral exposure to these foods.

### Histopathologic findings

**Fig 2:**
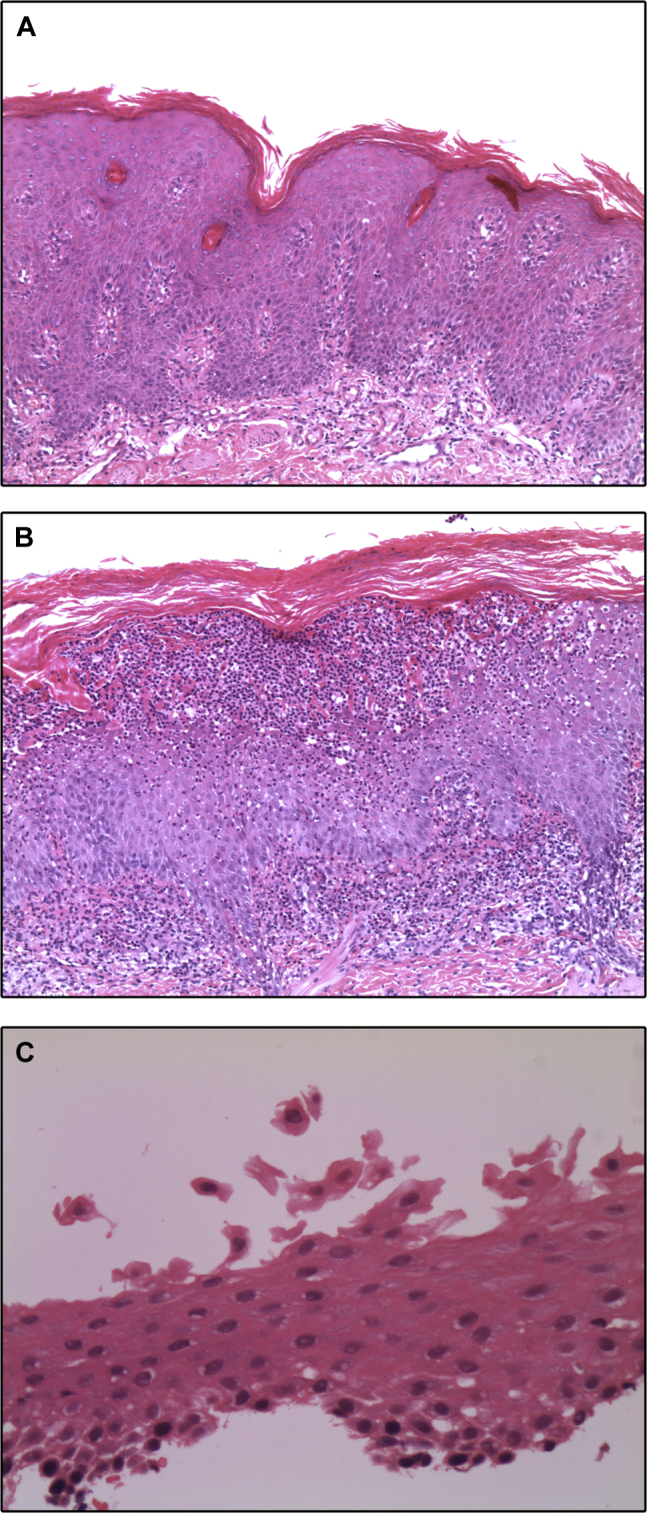
Histopathology. **A,** Skin showing irregular psoriasiform hyperplasia, hyperkeratosis and parakeratosis, and a mild superficial dermal inflammatory infiltrate. **B,** Skin showing a florid subcorneal pustular dermatosis superimposed on background changes similar to those in Fig 2, *A*, with irregular hyperplasia and marked hyperkeratosis and parakeratosis. **C,** Esophageal squamous mucosa showing separation and detachment of superficial squamous epithelial cells in the absence of any mucosal inflammatory process.

Skin biopsies performed when the patient was 3 and 16 months of age showed similar findings (Fig 2, *A*). There was hyperkeratosis and parakeratosis overlying an acanthotic epidermis. The granular layer was absent, and there was no evidence of acantholysis. A superficial dermal inflammatory infiltrate composed of lymphocytes, admixed neutrophils, and histiocytes was present (Fig 2, *B*). A skin biopsy of the pustular eruption performed when the patient was 26 months of age showed a florid pustular dermatosis superimposed on the previously reported histologic findings (Fig 2, *B*). The pustule was intraepidermal and restricted to the stratum corneum. Hair microscopy was noncontributory, and notably, there was no trichorrhexis invaginata. Esophageal biopsies were done when the patient was 10 and 16 months old. They showed separation, detachment, and acantholytic sloughing of the superficial squamous epithelial cells in the absence of any significant inflammatory process or spongiosis (Fig 2, *C*).

### Molecular genetics analysis

Whole-exome sequencing (WES) was performed in the proband with the Agilent SureSelect v4 capture kit (Agilent Technologies, Santa Clara, Calif) and sequenced on an Illumina HiSeq 2000 (Illumina, San Diego, Calif; GenePool, Edinburgh, United Kingdom). The resulting 88.7M 100-bp PE sequencing reads were aligned to the human genome (Ensembl release 68) with Bowtie2 (version 2.02; 98.8% aligned).^9^ Read duplicates were removed with Picard Tools (version 1.79; http://picard.sourceforge.net), and variants were called by using the UnifiedGenotyper in GATK-lite (version 2.2-8) according to the GATK best practices.^10^ The 69,732 called variants were annotated with the Variant Effect Predictor (version 72),^11^ and any variants with the following consequences were filtered out: downstream_gene_variant, upstream_gene_variant, synonymous_variant, intergenic_variant, and intron_variant. Filtered data were put in a MySQL database, allowing querying through a Django interface. The desmoplakin variant p.His586Pro was confirmed by means of Sanger sequencing with the primers and conditions previously reported for exon 14 of the *DSP* gene.^12^ Array comparative genome hybridization (aCGH) was carried out with the Cytoscan 750K high-density oligo array (Affymetrix UK, High Wycombe, United Kingdom), according to the manufacturer's recommended conditions (analysis performed under contract by the South East Scotland Cytogenetics Laboratory, Western General Hospital, Edinburgh, United Kingdom).

### Ultrastructure

Skin biopsy specimens were prefixed in half-strength Karnovsky fixative, followed by postfixation in reduced 1% OsO_4_ containing 1.5% potassium ferrocyanide. After embedding in an Epon epoxy resin, ultrathin sections (600 Å) were mounted on Formvar-coated grids, counterstained with uranyl acetate and lead nitrate, and examined in a Jeol JEM 100 CX electron microscope (60 kV).

### Immunohistochemistry of skin biopsy specimens

Four-micrometer paraffin-embedded sections were baked overnight at 60°C and deparaffinized with xylene, followed by a gradient of ethanol washes. Sections were permeabilized with 0.5% Triton X-100. Antigen retrieval was done by heating sections to 95°C for 15 minutes in 0.01 mol/L citrate buffer (pH 6.0). Slides were blocked in 1% BSA and 2% normal goat serum and incubated for 1 hour at 37°C. Primary antibodies were diluted in 1% BSA and 2% normal goat serum and incubated overnight at +4°C. Mouse mAbs used were as follows: 4B2 (desmoglein 1; diluted 1:100)^13^ and 115F (desmoplakin, a gift from David Garrod; diluted 1:100). Polyclonal antibodies used were as follows: keratin 10 (a gift from Julie Segre; diluted 1:2000) and 1407 (plakoglobin, diluted 1:2000). Secondary staining was done with Alexa Fluor–conjugated antibodies to mouse, rabbit, or chicken (Life Technologies, Grand Island, NY) diluted 1:300 in 1% BSA and 2% normal goat serum for 30 minutes at 37°C. All washes were done with PBS. Coverslips were mounted with polyvinyl alcohol for microscopic analysis by using a DMR Leica microscope (×40 NA 1.0 Plan-Fluotar Plan-Apochromat objective) and a charge-coupled device camera (Orca 100 model CA7 42-95; Hamamatsu Photonics, Hamamatsu City, Japan). 4′,6-Diamidino-2-phenylindole was added during the secondary antibody incubation at a final concentration of 2 μg/mL.

## Results

### Molecular genetics

Initial mutation screening of the proband's genomic DNA isolated from peripheral blood was unremarkable. This included Sanger sequencing of the entire coding sequences of *SPINK5* and ADAM metallopeptidase domain 17 *(ADAM17)*, as well as 8 genes known to cause autosomal recessive congenital ichthyosis *(NIPAL4*, *CYP4F22*, *ALOXE3*, *ALOXE12B*, *TGM1*, *PNPLA1*, *ABCA12*, *CERS3*, *FATP4*, and *ABHD5)* and pustular psoriasis (*CARD14* and *IL36RN)*. Results of DNA repair studies were entirely normal. The patient had a normal male karyotype, and aCGH showed no major deletions or duplications.

The proband's DNA was then subjected to WES. Given the known association of *DSG1* mutations in prior cases of SAM syndrome, we screened WES variant data for mutations in *DSG1* and related desmosomal genes. Repetitive analysis was initially focused on the *DSG1* gene, which was previously reported to be causative in patients with SAM syndrome. No potentially pathogenic variants were identified in the *DSG1* gene, and therefore genes encoding other desmosomal proteins were examined. Only nonpathogenic single nucleotide polymorphisms (SNPs) that have been found in healthy populations by the 1000 Genomes Project were identified in the desmosomal protein genes listed in Table I, with the exception of the novel point mutation c.1757A>C in *DSP* (encoding desmoplakin) predicting the amino acid substitution mutation p.His586Pro in the desmoplakin polypeptide.

**Fig 3:**
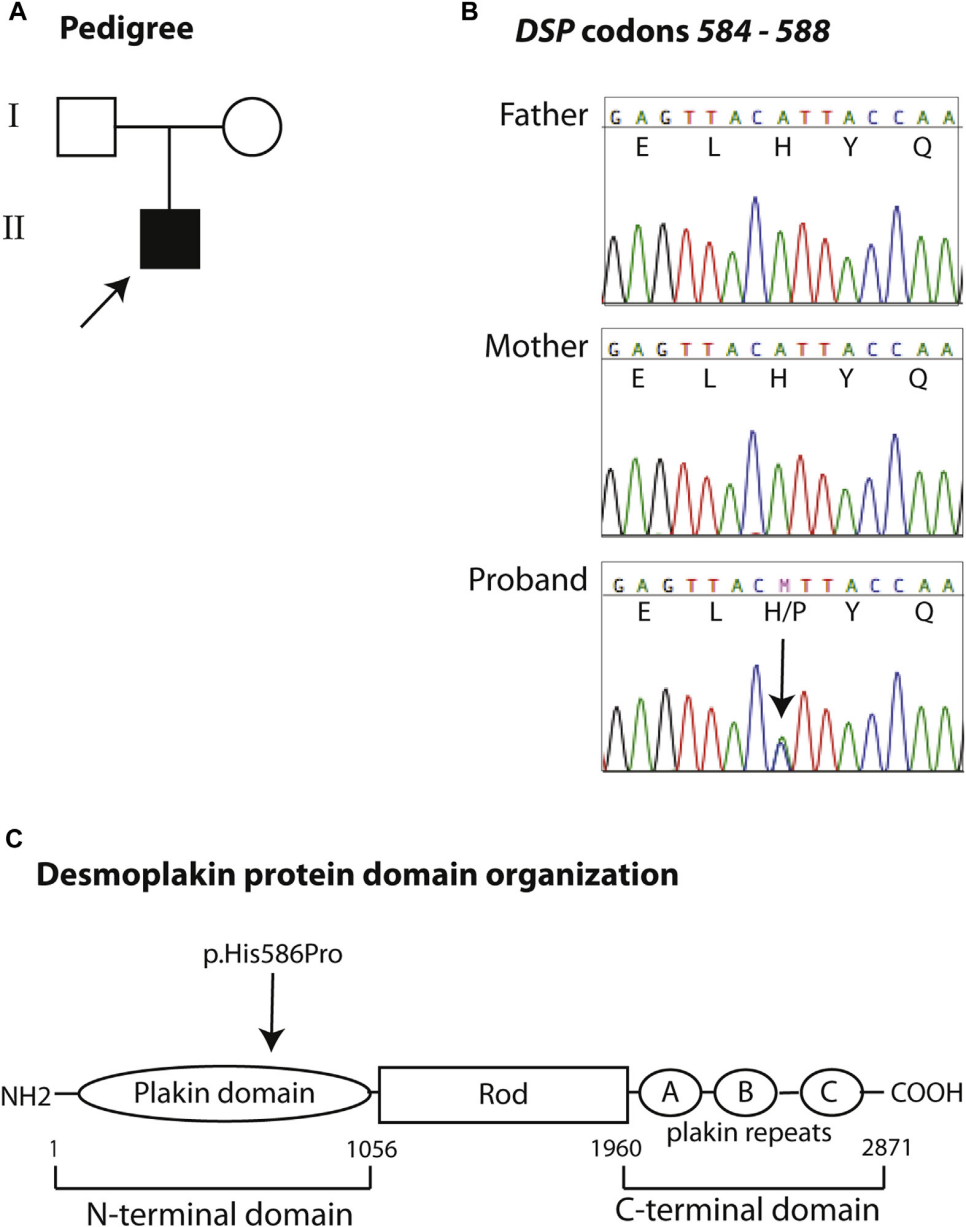
Molecular genetics. **A,** Pedigree of the family in this study. The *arrow* indicates the proband. **B,** Sequence of the *DSP* exon 14 spanning codons 584 to 588. Both parents are wild-type for this region, and the proband has the heterozygous missense mutation c.1757A>C (indicated by the *arrow*), predicting the amino acid substitution p.His586Pro at the protein level. **C,** Primary structure of desmoplakin. Amino acid boundaries of the 3 major domains of the protein are indicated. The p.His586Pro mutation lies within the plakin domain in the N-terminal head of desmoplakin.

The mutation was confirmed by means of Sanger sequencing (Fig 3, *A* and *B*) and excluded from 100 unrelated, ethnically matched healthy control subjects (200 alleles) by using Sanger sequencing. The mutation was not detected in the proband's parents, indicating that it represents a *de novo* change. This mutation lies within the plakin domain of desmoplakin, which is shared by other members of the plakin protein family (Fig 3, *C*). The crystal structure of residues 175 to 630 of desmoplakin was described by Choi and Weis.^14^ Histidine 586 is located in an α-helix within spectrin repeat 6, a structure that is conserved in bullous pemphigoid antigen 1 and plectin.^14^ Histidine is a hydrophilic charged amino acid, whereas proline is a hydrophobic residue, and therefore this is a nonconservative amino acid substitution. More importantly, proline introduces a strong turn structure within polypeptide chains, and therefore this variant is strongly predicted to completely disrupt the conserved α-helix within desmoplakin's spectrin repeat 6.^15^, ^16^

As an incidental finding, both the proband and his father were heterozygous for a c.6208G>A missense SNP in *DSP*, predicting the protein change p.Asp2070Asn (Table I). This SNP (rs41302885) was absent in maternal DNA. SNP rs41302885 has been found at a low frequency in healthy populations, with minor allele frequencies of 0.0026 (1000 Genomes Project; http://www.ncbi.nlm.nih.gov/SNP/) and 0.0033 (Exome Variant Server; http://evs.gs.washington.edu). Therefore this variant, like those seen in other desmosomal genes (Table I), is predicted to be a nonpathogenic polymorphism.

High-density aCGH was performed to exclude the presence of a second compound heterozygous genomic mutation that might be missed by both WES and targeted PCR-based sequencing, such as deletion or duplication of 1 or more exons. The Affymetrix 750K array used had full probe coverage for all exons of the *DSP* and *DSG1* genes. No copy number changes were observed for either gene by using high-density aCGH. Consistent with this result, the coverage of WES sequence reads across the *DSP* gene was not significantly different from that of matched control samples (data not shown). Thus all molecular genetics data are consistent with the *de novo* missense mutation p.His586Pro in *DSP* being the causative genetic lesion.

### Ultrastructural analysis

**Fig 4:**
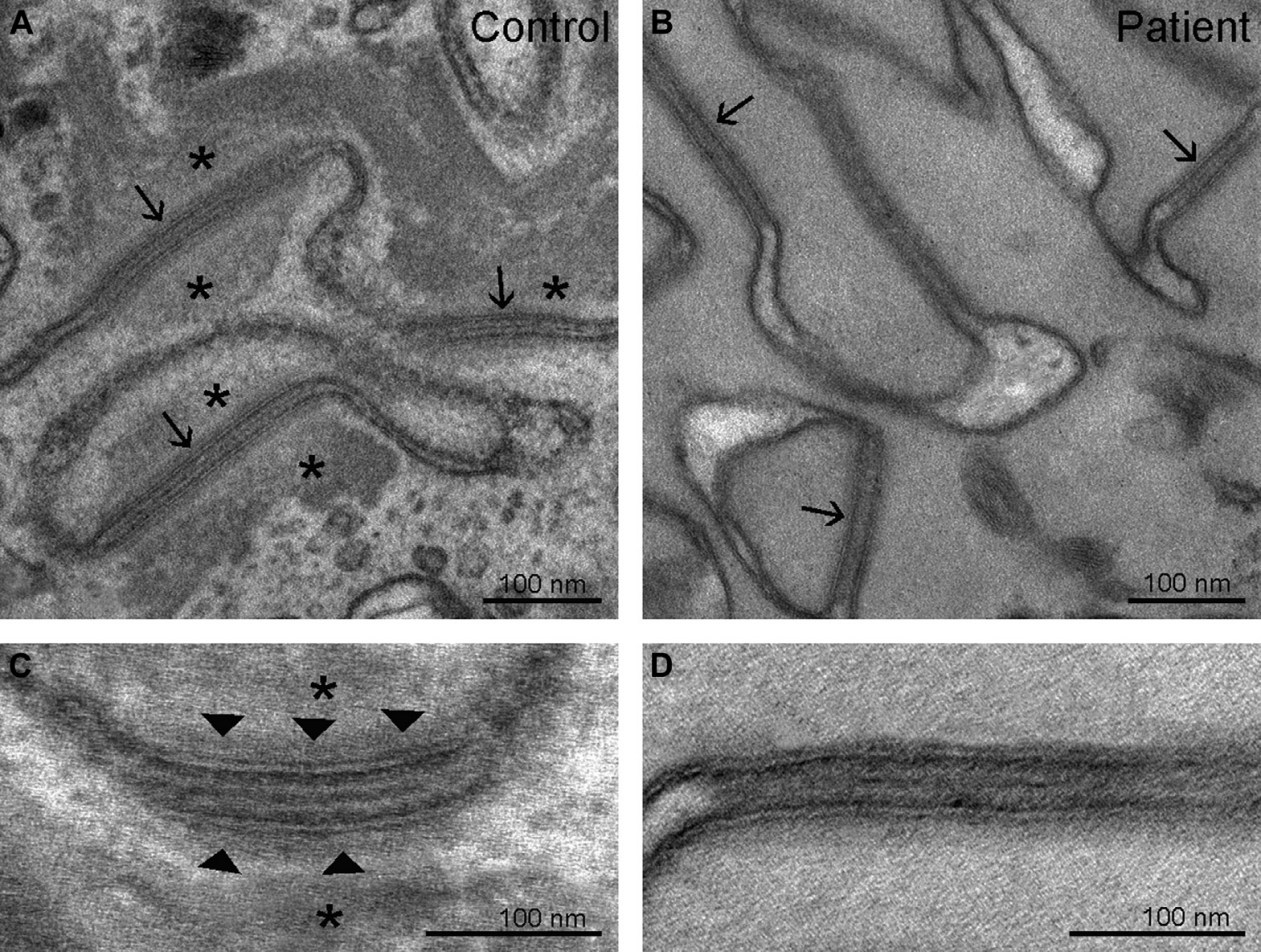
Ultrastructural abnormalities. **A,** Abundant keratin filaments *(asterisks*) anchored to desmosomes *(arrows)* in the cytoplasm of a spinous cell in normal human epidermis. **B,** Note the scarcity of keratin filaments at the same level in the proband. **C,** Common desmosomal structure with normal inner dense plaque *(arrowheads)* in a human control sample. **D,** In contrast, a poorly formed inner dense plaque lacking evidence of keratin filament attachment was observed in the proband.

**Fig 5:**
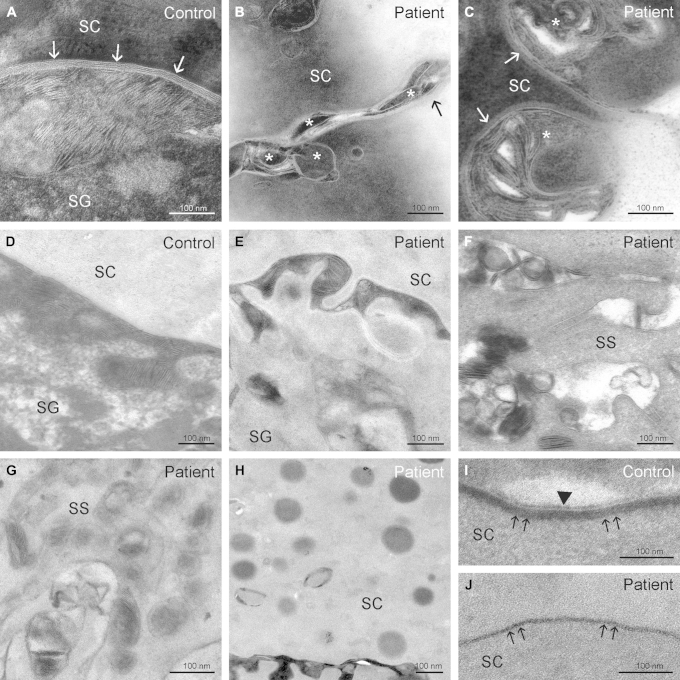
Stratum corneum *(SC)* ultrastructural abnormalities. **A,** Mature lamellar bilayers *(arrows)* and normal postsecretory lipid processing in the upper portion of the stratum granulosum *(SG)*–SC interface in normal human skin. **B** and **C**, In addition to areas of processed lamellar bilayer arrays *(arrows)*, the patient sample shows abnormal lamellar bilayer organization with disruption by nonlamellar domains (Fig 5, *B*, *asterisks*), delayed lipid processing, and incompletely processed lamellar material (Fig 5, *C*, *arrows* and *asterisks*, respectively). **D,** Normal lamellar body secretion with homogenous extracellular bilayers in a healthy human. **E** and **F**, Inhomogenous lamellar body secretion with foci of vesicular contents at the SG-SC interface and premature secretion in the stratum spinosum *(SS)* in the patient. **G,** Predominantly abnormal, ellipsis-shaped lamellar bodies with aberrant internal structures in the patient. **H-J,** Non–membrane-bound droplets throughout the SC, containing electron-dense material, possibly lipids (Fig 5, *H*). In contrast to the normal regular cornified envelope *(double arrows)* and corneocyte lipid envelope (Fig 5, *I*, *arrowhead*), note the thinning of the cornified envelope *(double arrows)* and absence of the corneocyte lipid envelop in the patient (Fig 5, *J*).

In normal human epidermis keratin filaments make looping attachments with the inner plaques of desmosomes.^17^ Electron microscopy of the patient's skin (Fig 4) showed a striking disassociation between keratin filament bundles (asterisks in the control tissue image) and desmosomes in the spinous layer of the epidermis, where desmosomes are most clearly visualized (Fig 4, *A* and *B*). The desmosomal inner plaques were much less electron dense and appeared poorly formed compared with desmosomes observed in normal control skin (Fig 4, *C* and *D*). The stratum corneum also displayed striking structural abnormalities (Fig 5), including marked attenuation of cornified envelopes (Fig 5, *J*, double arrows), a virtual absence of corneodesmosomes, and abnormalities in the postsecretory maturation (Fig 5, *C*) and organization of secreted lamellar body contents (Fig 5, *B*, and *E-G*).

### Immunohistochemistry

**Fig 6:**
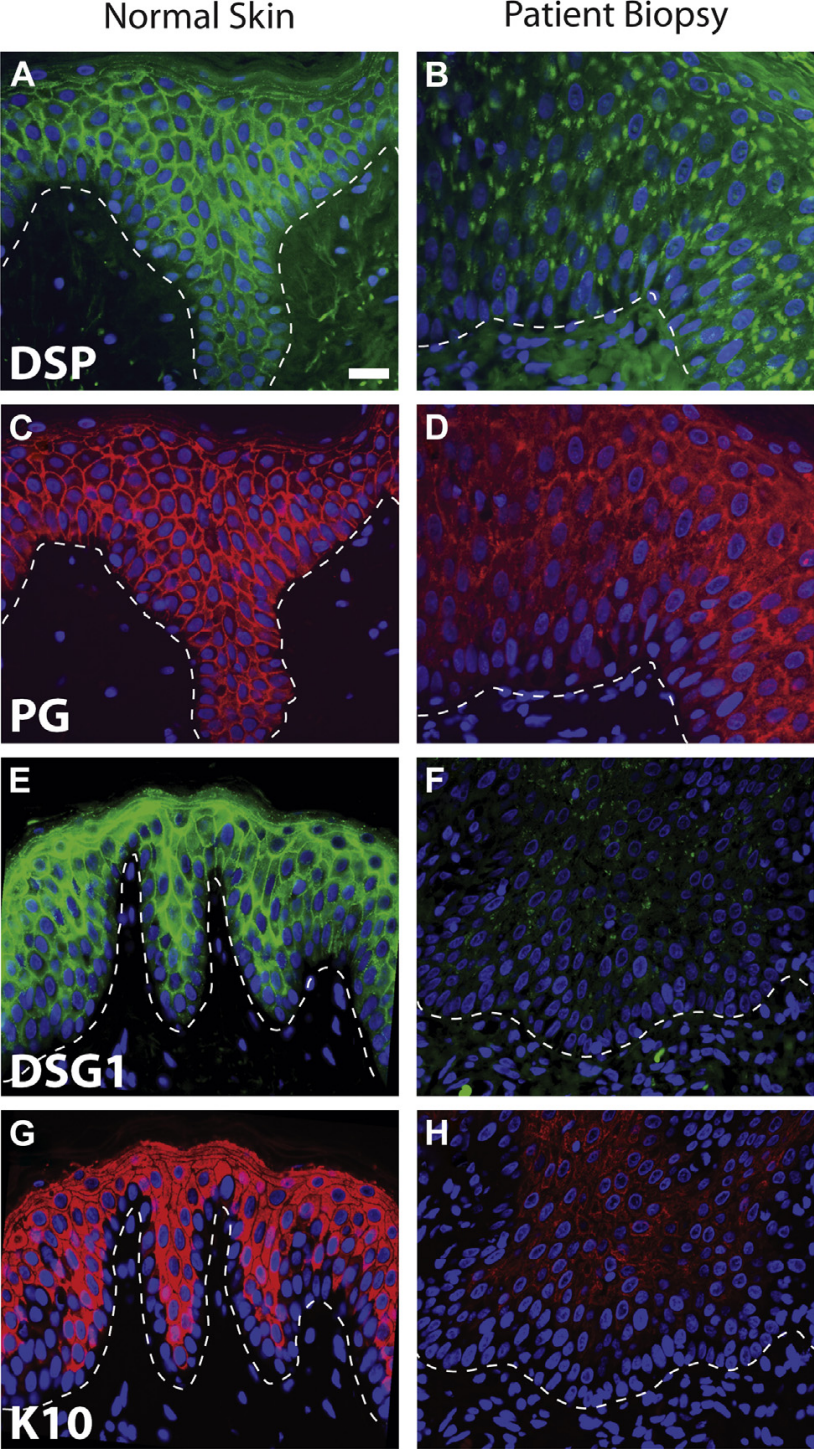
Immunostaining. Paraffin-embedded sections of control biopsy material from patients and control subjects were stained for key desmosome components and interacting epidermal proteins (control epidermis: **A, C, E,** and **G,***left panels*; proband: **B, D, F,** and **H,***right panels*). Fig 6, *A* and *B*, Staining for desmoplakin (*DSP*; *green*). Note the reduction in staining in the proband's skin plus the accumulation of desmoplakin in the cytoplasm. Fig 6, *C* and *D*, Plakoglobin staining (*PG*; *red*) is weaker in the patient's skin and appears less intense at the cell borders. Fig 6, *E* and *F*, Desmoglein 1 expression (*DSG1*; *green*) is drastically reduced in the patient's epidermis. Fig 6, *G* and *H*, Staining for keratin 10 *(K10)*, a major component of the intermediate filament cytoskeleton in suprabasal keratinocytes, which was greatly reduced in the proband. Nuclei were visualized with 4′,6-diamidino-2-phenylindole. The *dashed line* in each image indicates the location of the dermal-epidermal junction. Calibration bar = 20 μm.

Immunofluorescence analysis revealed aberrant desmoplakin staining, appearing as large accumulations or aggregates (Fig 6, *A* and *B*). Striking reductions in both desmoglein 1 and keratin 10 staining were observed (Fig 6, *E-H*). What *DSG1* remained appeared in aggregates. Plakoglobin distribution appeared less perturbed, with largely plasma membrane–associated staining punctuated by fluorescence-bright regions that colocalized in some cases with desmoplakin aggregates (Fig 6, *C* and *D*).

## Discussion

Intercellular junction complexes are a diverse group of organelles that function to provide adherence and communication between individual cells, as well as contributing to the integrity of larger tissues. These intercellular junctions include desmosomes, adherens junctions, tight junctions, and gap junctions.^18^ Desmosomes are intercellular attachment and anchoring sites for the intermediate filament cytoskeleton.^19^ Electron microscopic studies have characterized their ultrastructure as containing the intermediate filament-associated inner plaque, an electron-dense outer plaque, the plasma membrane, and the electron-dense midline in the extracellular domain.^18^

Desmosomes are composed of several transmembrane and intracellular molecules. The transmembrane proteins facilitating intercellular adhesion are desmosomal cadherins, desmogleins, and desmocollins.^17^ Intracellular domains are attached to the intermediate filaments through a range of linker molecules, including desmoplakin, plakoglobin, and plakophilins.^17^

Desmosomal structures are widely expressed in epithelia, including the intestinal mucosa, gall bladder, uterus and oviduct, liver, pancreas, stomach, salivary and thyroid glands, and epithelial cells of the nephron, but they are most abundant in tissues that experience mechanical stress, such as the skin and myocardium.^18^ Not only do desmosomes provide resistance to the strong mechanical forces applied to skin and cardiac muscle and therefore contribute to tissue cohesiveness, they also have a role in cell signaling and skin barrier functionality.^17^, ^19^ These important functions explain, in part, the diverse range of disease phenotypes observed in patients with desmosomal diseases. Genetic abnormalities in desmosomal components result in a variety of skin and cardiac diseases.

Desmoplakin is an obligate component of functional desmosomes and is highly expressed in the heart, epidermis,^18^, ^19^ and hair follicles.^20^ Desmoplakin associates with intermediate filaments through its C-terminus and interacts with plakophilins and plakoglobin through its N-terminus to target the desmoplakin-keratin complex to the desmosomal inner plaque.^21^, ^22^ Desmoplakin haploinsufficiency has been reported to cause autosomal dominant type II striate PPK without any other skin, hair, or extracutaneous features.^23^ This clinical phenotype suggests that a 50% expression level of desmoplakin is sufficient for epidermal functioning in most body sites but not for the palms and soles, which are subject to considerable mechanical stress.^17^ Recessive mutations in desmoplakin result in severe phenotypes. Carvajal syndrome is characterized by striate PPK, woolly hair, and left ventricular cardiomyopathy.^24^ Skin fragility/woolly hair syndrome is also caused by recessive mutations in *DSP*. Reported cases included compound heterozygosity for a nonsense/missense combination of mutations. The clinical phenotype described in the reports was a focal and diffuse PPK, hyperkeratotic plaques on the trunk and limbs, and varying degrees of alopecia but no apparent cardiomyopathy. Heterozygous carriers of these mutations displayed no phenotypic abnormalities.^12^ Lethal acantholytic epidermolysis bullosa has been reported in a neonate presenting as complete alopecia, neonatal teeth, nail loss, extensive skin erosion, and neonatal death. The infant had compound heterozygosity for a recessive nonsense and frameshift *DSP* mutation, resulting in deletion of the intermediate filament-binding sites in the desmoplakin tail domain.^25^

Our case is the first reported case of SAM syndrome caused by a *DSP* mutation. The recently reported families with SAM syndrome had biallelic loss-of-function mutations in *DSG1*.^7^, ^8^ Desmoglein 1 is a member of the desmosomal cadherins and strongly expressed in the granular and spinous layers of the epidermis^18^, ^19^ and hair follicles.^20^ Heterozygous mutations in *DSG1* cause type I striate PPK. Homozygous *DSG1* mutations were reported to cause the severe phenotype of SAM syndrome.^7^ The first reported cases were from 2 families with consanguineous healthy parents.^7^, ^8^ Their described phenotype was congenital erythroderma; skin erosions and scaling; yellowish papules and plaques at the periphery of the palms, along the fingers, and over weight-bearing areas of the feet; and hypotrichosis. From early infancy, they had markedly increased IgE levels, severe food allergies, and recurrent infections with severe metabolic wasting.^7^ Minor cardiac developmental defects were noted in 2 patients, which is in keeping with the association between desmosomal diseases and cardiac conditions.^7^ Two of the patients also had esophageal involvement, which was similar to our patient.^7^ The fourth reported case, the only child of healthy unrelated parents, had a milder phenotype with PPK, dermatitis, and multiple allergies but had normal hair and was otherwise well.^8^ Samuelov et al^7^ demonstrated that *DSG1* deficiency was associated with increased expression of genes encoding the allergy-related cytokines thymic stromal lymphopoietin, IL-5, and TNF. It is notable that our patient and other patients with SAM syndrome had esophageal involvement; our patient had separation and detachment of the esophageal superficial squamous cells, and another patient with SAM syndrome had typical eosinophilic esophagitis.^7^ Similar to SAM syndrome, eosinophilic esophagitis is characterized by allergic inflammation of the esophageal mucosa, immune sensitization to foods, and impaired esophageal barrier function.^26^ Recent data have suggested a functional role for *DSG1* and its dysregulation in the pathophysiology of eosinophilic esophagitis. Furthermore, the loss of *DSG1* expression might potentiate allergic inflammation through induction of proinflammatory mediators.^26^

Our patient showed a marked reduction in desmoglein 1 protein expression, as shown by immunohistochemical staining of the skin (Fig 6), despite not having a *DSG1* mutation. This reduction in expression might be due, at least in part, to disruption of stable complexes comprising both *DSP* and *DSG1*. The primary structure of desmoplakin has 3 distinct regions: the 1056-amino-acid N-terminal domain, an 890-residue central coiled-coil domain, and a 925-residue C-terminal intermediate filament binding domain (Fig 3, *C*).^14^ Yeast two-hybrid assays and coimmunoprecipitation experiments showed that desmoplakin's N-terminal 584-amino-acid region is necessary and sufficient to target desmoplakin to the inner desmosomal plaque through its association with armadillo proteins.^14^, ^21^ It is likely that replacement of histidine with proline in close proximity to this region disrupts protein conformation and possibly armadillo protein binding, leading to defects in *DSP* localization and function. More recently, the microtubule plus tip protein end-binding protein 1 was identified as a binding partner for this same region of desmoplakin.^27^ In addition, the observed loss of *DSG1* that occurs secondary to desmoplakin defects might be a causative factor in decreasing keratin expression because *DSG1* suppresses the extracellular signal–regulated kinase signaling required to promote differentiation, including expression of genes important for barrier formation.^28^, ^29^ Thus *DSG1* not only maintains adhesion in the upper epidermis but also instructs an early epidermal differentiation program on stratification.^29^ The attenuation of differentiation might also lead to loss of expression of other epidermal proteins, therefore further exacerbating the barrier defect. Because *DSG1* expression or localization is affected in all cases of SAM syndrome, it is unclear whether it is the loss of *DSG1* or *DSP* at intercellular junctions that might be causative in the disease.

Although *FLG* mutations are the most significant mutations associated with AD, many other genes involved in skin barrier function have been implicated, including SNPs in the *SPINK5* gene.^30^, ^31^ Homozygous or compound heterozygous loss-of-function mutations in the *SPINK5* gene result in the autosomal recessive disorder Netherton syndrome, with severe AD and allergy.^1^ Furthermore, proteomic profiling of skin from patients with AD has found that multiple other proteins related to the skin barrier were expressed at significantly lower levels in lesional compared with nonlesional sites of patients with AD. These proteins included filaggrin 2, corneodesmosin, desmoglein 1, desmocollin 1, and transglutaminase 3.^30^ Nonlesional AD skin might also partially share the lesional skin phenotype. It has been shown that nonlesional skin has reduced expression of filaggrin and filaggrin-like proteins,^32^ as well as increased expression of immune genes.^33^

The pathomechanisms of AD are complex and include interplay between epidermal structural abnormalities and immune dysregulation. However, there are several lines of evidence supporting the role of an aberrant skin barrier in the development of atopic diseases and allergy. The compromised skin barrier might allow enhanced allergen exposure to the cutaneous immune system, enhanced T_H_2 responses, and development of allergies. Alternatively, or in addition, the structural protein-deficient epithelium might be proinflammatory and primed for the development of allergy.^34^

We report a fifth case of SAM syndrome and the first case caused by desmoplakin mutations. SAM syndrome substantiates the role of heritable skin barrier defects, particularly in desmosomal proteins, in the pathogenesis of atopic disease. The pathomechanisms of SAM syndrome require further investigation and might provide valuable insights into the development of atopic diseases.

**Clinical implications**

Three families with SAM syndrome have been reported, all with mutations in *DSG1*. We report a fifth case, the first linked to a *DSP* mutation. In patients with SAM syndrome, mutations in *DSG1*, *DSP*, and other desmosomal proteins should be considered.

## Figures and Tables

**Table I:** Desmosome protein variants

Gene	Protein	Missense SNP	Zygosity	dbSNP no.	Minor allele frequency (1000 Genomes Project)
*DSP*	Desmoplakin	p.Asp2070N	Het	rs41302885	0.0026
		**p.His586Pro**	**Het**	**None**	**None**
*DSG1*	Desmoglein 1	p.Met11Val	Hom	rs1426310	0.3626
*DSG2*	Desmoglein 2	p.Ile293Val	Het	rs2230234	0.0323
		p.Arg773Lys	Het	rs2278792	0.2400
*DSG3*	Desmoglein 3	p.Thr912Ala	Hom	rs1380866	0.0008
*DSG4*	Desmoglein 4	p.Ile644Leu	Hom	rs4799570	0.0341
*DSC1*	Desmocollin 1	p.Met659Thr	Het	rs28620831	0.0220
		p.Cys848Phe	Het	rs985861	0.0933
*DSC2*	Desmocollin 2	p.Ile776Val	Het	rs1893963	0.1965
*DSC3*	Desmocollin 3	None			
*JUP*	Plakoglobin	p.Arg142His	Het	rs41283425	0.0240
		p.Met697Leu	Hom	rs1126821	0.4167
*PKP1*	Plakophilin 1	None			
*PKP2*	Plakophilin 2	None			
*PKP3*	Plakophilin 3	p.Gly95Arg	Het	rs11246148	0.3608
*PKP4*	Plakophilin 4	None			
*CSDN*	Corneodesmosin	None			
*EVPL*	Envoplakin	p.Gln433Arg	Het	rs2071192	0.3241
*EVPLL*	Envoplakin-like	p.Ser4Asn	Het	rs570145	0.4766
*PPL*	Periplakin	p.Arg819Ser	Hom	rs2734742	0.0617
		p.Ala1007Val	Het	rs2075639	0.1194

*Het*, Heterozygous; *Hom*, homozygous.

Variables in boldface are disease causing.
